# Development and validation of a PCR-free nucleic acid testing method for RNA viruses based on linear molecular beacon probes

**DOI:** 10.1186/s12951-022-01470-1

**Published:** 2022-06-11

**Authors:** Fuyu Du, Weijie Zhang, Huimin Yao, Yuqiong Xia, Xianghan Zhang, Peng Yang, Pengbo Ning

**Affiliations:** 1 School of Life Science and Technology, Xidian University, Xi’an, 710071 Shaanxi People’s Republic of China; 2 Engineering Research Center of Molecular & Neuroimaging, Ministry of Education, Xi’an, 710071 Shaanxi People’s Republic of China

**Keywords:** SARS-CoV-2, CSFV, Nucleic acid testing, PCR-free, Molecular beacon probes

## Abstract

**Background:**

RNA viruses periodically trigger pandemics of severe human diseases, frequently causing enormous economic losses. Here, a nucleic acid extraction-free and amplification-free RNA virus testing probe was proposed for the sensitive and simple detection of classical swine fever virus (CSFV) and severe acute respiratory syndrome coronavirus 2 (SARS-CoV-2), based on a double-stranded molecular beacon method. This RNA virus probe contains two base sequences—a recognition strand that binds to the specific domain of CSFV N2 or SARS-CoV-2 N, with a fluorophore (FAM) labeled at the 5′ end, and a complementary strand (CSFV-Probe B or SARS-CoV-2-Probe B), combined with a quencher (BHQ2) labeled at the 3′ end.

**Results:**

Using linear molecular beacon probe technology, the detection limit of the RNA virus probe corresponding to CSFV and SARS-CoV-2 were as low as 0.28 nM and 0.24 nM, respectively. After CSFV E2 and SARS-CoV-2 N genes were transfected into corresponding host cells, the monitoring of RNA virus probes showed that fluorescence signals were dramatically enhanced in a concentration- and time-dependent manner. These results were supported by those of quantitative (qRT-PCR) and visualization (confocal microscopy) analyses. Furthermore, CSF-positive swine samples and simulated SARS-CoV-2 infected mouse samples were used to demonstrate their applicability for different distributions of viral nucleic acids in series tissues.

**Conclusions:**

The proposed RNA virus probe could be used as a PCR-free, cost-effective, and rapid point-of-care (POC) diagnostic platform for target RNA virus detection, holding great potential for the convenient monitoring of different RNA viruses for early mass virus screening.

**Graphical Abstract:**

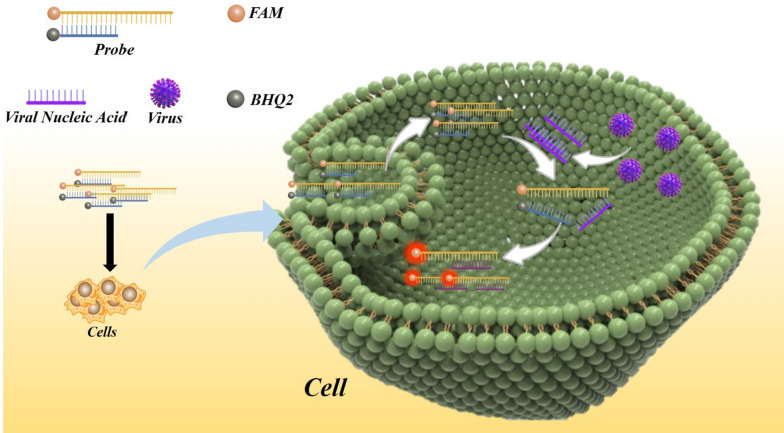

**Supplementary Information:**

The online version contains supplementary material available at 10.1186/s12951-022-01470-1.

## Introduction

**Fig. 1:**
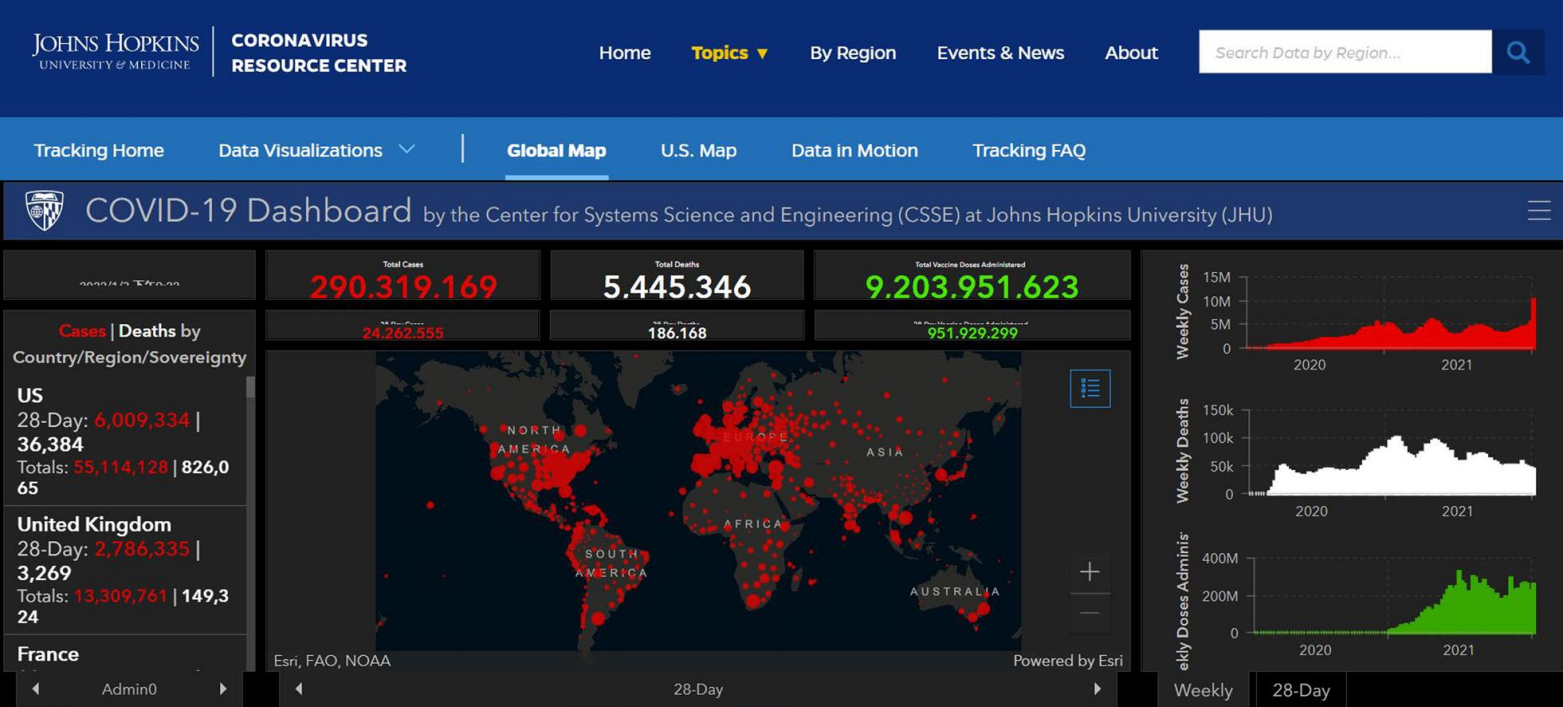
The Johns Hopkins Coronavirus Resource Center webpage. This webpage “Global Map” was obtained from its website (https://coronavirus.jhu.edu/map.html) in January 2022. COVID-19 cases and death were tracked and determined in real time around the world. More comprehensive information of one country or territory could be acquired from the Johns Hopkins Coronavirus Resource Center webpage, which could offer other available information in regards to the continued pandemic

RNA viruses have periodically triggered a series of severe human diseases, including Ebola hemorrhagic fever, severe acute respiratory syndrome (SARS), Middle East respiratory syndrome (MERS), influenza, hepatitis C, acquired immune deficiency syndrome (AIDS), and coronavirus disease 19 (COVID-19). Additionally, RNA viruses such as classical swine fever virus (CSFV) frequently cause enormous economic losses in the global animal breeding industry [1]. Therefore, early diagnosis of viral diseases is critical for the prevention and control of subsequent worldwide pandemics. The novel SARS coronavirus 2 (SARS-CoV-2) is currently prevalent, and as of January 2022, there were over 290.3 million and 5.4 million confirmed cases and deaths, respectively, attributed to COVID-19 across numbers of countries and territories worldwide (Fig. 1, Johns Hopkins University Coronavirus Resource Center, 2021, https://coronavirus.jhu.edu/map.html.). The fatality rate of SARS-CoV-2 infection is approximately 1–3%. COVID-19 is hazardous not only for the elderly, but also for middle-aged adults, for whom the risk of fatality from COVID-19 is higher than from an automobile accident or seasonal influenza [2, 3]. Although numerous available COVID-19 diagnostic products have helped to combat the current viral pandemic, the need to facilitate efficient and large-scale population screening remains [4, 5].

Immunodiagnostic methods, such as ELISA [6], utilize a series of classic virus detection techniques involving viral proteins and specific antibodies. However, even without considering the long cycle or high cost of antibody preparation, sensitivity is still a challenge for many immunology diagnostic technology platforms. Some novel nanotechnologies are being used for point-of-care testing on SARS-COV-2 targets, but it often have to rely on expensive antibodies and complicated process preparation [7]. Consequently, nucleic acid-based diagnostic methods, such as reverse-transcriptase polymerase chain reaction (RT-PCR), considered the gold standard for the diagnosis of RNA viruses, are recommended by the World Health Organization (WHO) [8]. However, these laboratory-based PCR techniques often require large volumes of expensive reagents and equipment and trained personnel to perform multiple time-consuming steps, such as nucleic acid extraction and amplification. Although the ongoing COVID-19 pandemic has produced an unparalleled need for rapid diagnostic testing, a major bottleneck to widespread SARS-CoV-2 testing is the RNA extraction step [9]. The improper extraction of nucleic acid from clinical materials is the primary cause of false-negative RT-PCR results. Additionally, transportation and storage conditions are essential in ensuring the stability of samples and generating accurate diagnostic results. A recent study reported that the rate of RNA degradation was significantly 5–10 fold higher in samples stored at 37 °C than at 20 °C, and even if stored at 20 °C versus 4 °C, the RNA degradation rate of samples increased 2–4 fold [10]. Furthermore, RNA degradation in samples affected the results of nucleic acid testing [11]. Some COVID-19 cases were initially reported as negative by SARS-CoV-2 RT-PCR and required multiple consecutive detections to obtain a positive result [12]. Such false negative results pose a great risk to epidemic prevention and control, because those false positive individuals may inadvertently spread infections in social activities. In livestock, the mixing of infected animals with healthy cattle can lead to devastating mass culls, thereby causing serious economic losses.

Considering the continued problems encountered during the detection of viral nucleic acids, it is essential to produce a field-ready sample-processing diagnostic strategy. Therefore, the present study aimed to design a novel nucleic acid extraction- and amplification-free RNA virus detection platform. As a proof of concept, we selected CSFV and SARS-CoV-2 as experimental models to calculate the accuracy of the double-stranded linear molecular beacon probes used in our method and prove that this direct method had sufficient sensitivity to facilitate nucleic acid testing of RNA viruses in living cells and animal samples.

## Results and discussion

### Design of an RNA virus molecular beacon probe

**Fig. 2:**
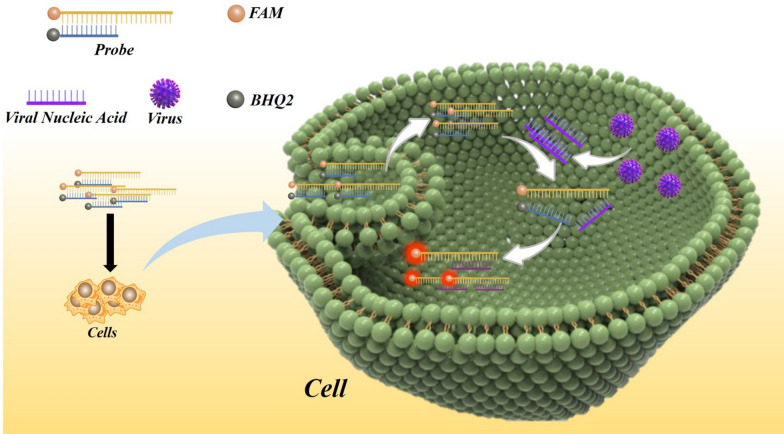
Scheme showing the mechanism of action of the fluorescent sensor for sequence-specific recognition of the target DNA. In the absence of the targeted RNA virus, this molecular beacon virus probe was in a “Signal off” state in which the fluorescence of the recognition strand was quenched due to fluorescence resonance energy transfer between the fluorophore (FAM) and the quencher (BHQ2). In the presence of RNA virus, it will competitively bind to the recognizing targeted virus nucleic acid and result in the release of the complementary strand, to induce the ‘‘Signal on’’ state of the fluorescence

Herein, we present the feasibility of a PCR-free strategy for the detection of RNA viruses. The principle of the molecular beacon virus probe is illustrated in Fig. 2. The probe consists of two strands; a recognition strand (CSFV-Probe A or SARS-CoV-2-Probe A) with a fluorophore (FAM) labeled at the 5′ end, and a complementary strand (CSFV-Probe B or SARS-CoV-2-Probe B) combined with a quencher (BHQ2) labeled at the 3′ end. As a proof of concept, the recognition strand was set as an RNA virus-recognizing sequence, in which the specific RNA sequence of CSFV E2 or SARS-CoV-2 N was employed. The complementary strand corresponds to a domain specific to the CSFV E2 or SARS-CoV-2 N sequence, and combines with the recognition strand by hybridization. In the absence of the targeted RNA virus, this molecular beacon virus probe remains in the “Signal off” state, in which the fluorescence of the recognition strand is quenched due to fluorescence resonance energy transfer between the fluorophore (FAM) and the quencher (BHQ2). However, in the presence of the target RNA virus, the recognitions strand will competitively bind to the recognizing targeted virus nucleic acid, resulting in the release of the complementary strand, to induce the ‘‘Signal on’’ state of fluorescence. Table 1 lists the sequences of the oligonucleotides used.

**Table 1:** Sequences of used oligonucleotides in this work (in 5′ to 3′ direction)

Type	Sequence
CSFV-Probe A	5′-FAM-GGATAGAGATTGGTTGATGATATTGCGTAC-3′
CSFV-Probe B	5′-CCAATCTCTAT-BHQ2-3′
Target-CSFV E2	5′-GTACGCAATATCATCAACCAAT-3′
SARS-CoV-2-Probe A	5′- FAM-GGATAGAGAATCTGTCAAGCAGCAGCAA-3′
SARS-CoV-2-Probe B	5′-ACAGATTCTCTAT-BHQ2-3′
Target-SARS-CoV-2 N	5′-TTGCTGCTGCTTGACAGATT-3′

### Optimization of RNA virus probe work concentration

**Fig. 3:**
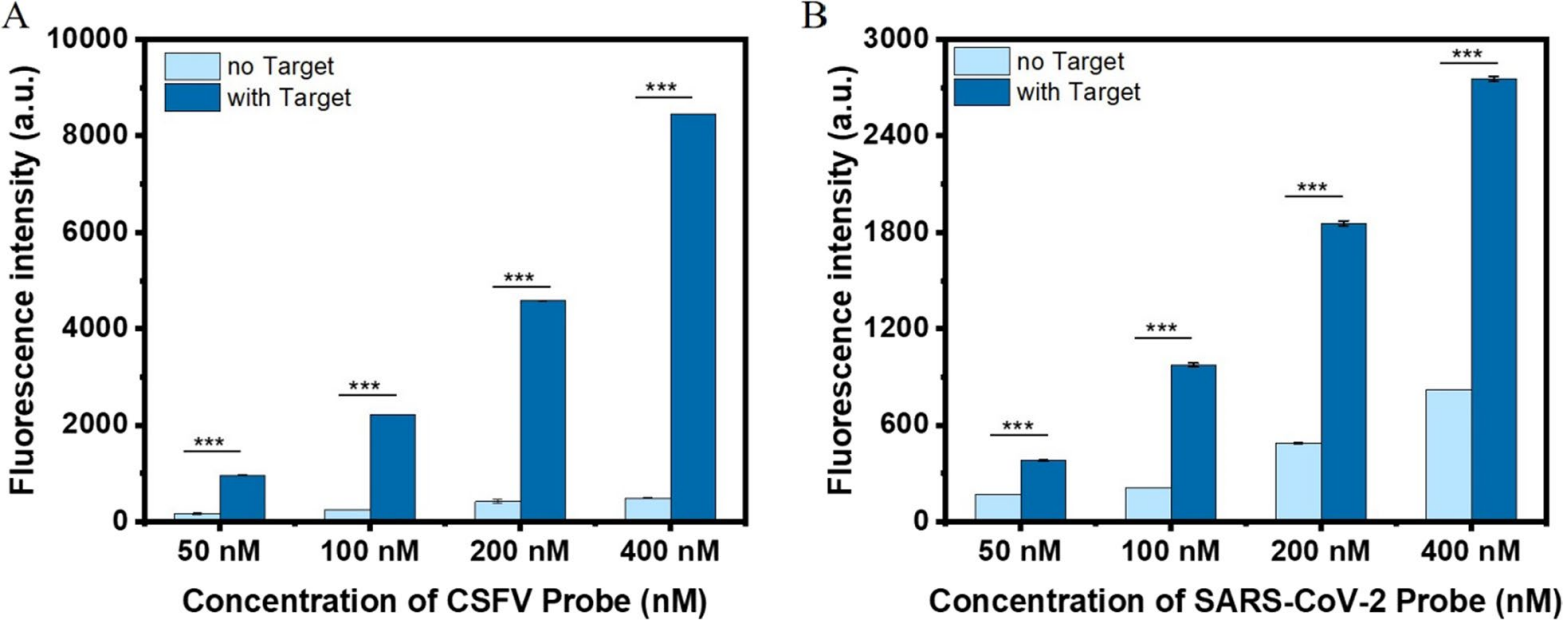
Optimization of the working concentration of CSFV and SARS-CoV-2 probes. **A** Fluorescence intensity of the assay on CSFV probe working concentration (50 nM, 100 nM, 200 nM and 400 nM). **B** Fluorescence intensity of the assay on SARS-CoV-2 probe working concentration (50 nM, 100 nM, 200 nM and 400 nM)

To establish the parameters for follow-up detection experiments, the optimum working concentration of the RNA virus probe was identified in a concentration gradient ranging from 50, 100, and 200 to 400 nM. As shown in Fig. 3, fluorescence intensity was dramatically enhanced when the target was mixed with an increased concentration of RNA virus probes. Qualitative analysis revealed that the fluorescence intensity was enhanced by approximately ten-fold when Target-CSFV E2 was added to the 100 nM probe solution (Fig. 3A). Meanwhile, the fluorescence intensity was enhanced by approximately five-fold when Target-SARS-CoV-2 N was mixed with a 100 nM probe (Fig. 3B). Thus, based on considerations of the significant test differences and future implementation costs, the probe working concentration was determined as 100 nM. The results of stability experiments support that the probe can work well under room temperature or physiological temperature (Additional file 1: Fig. S1).

### Sensitivity and specificity assay of RNA virus probe

**Fig. 4:**
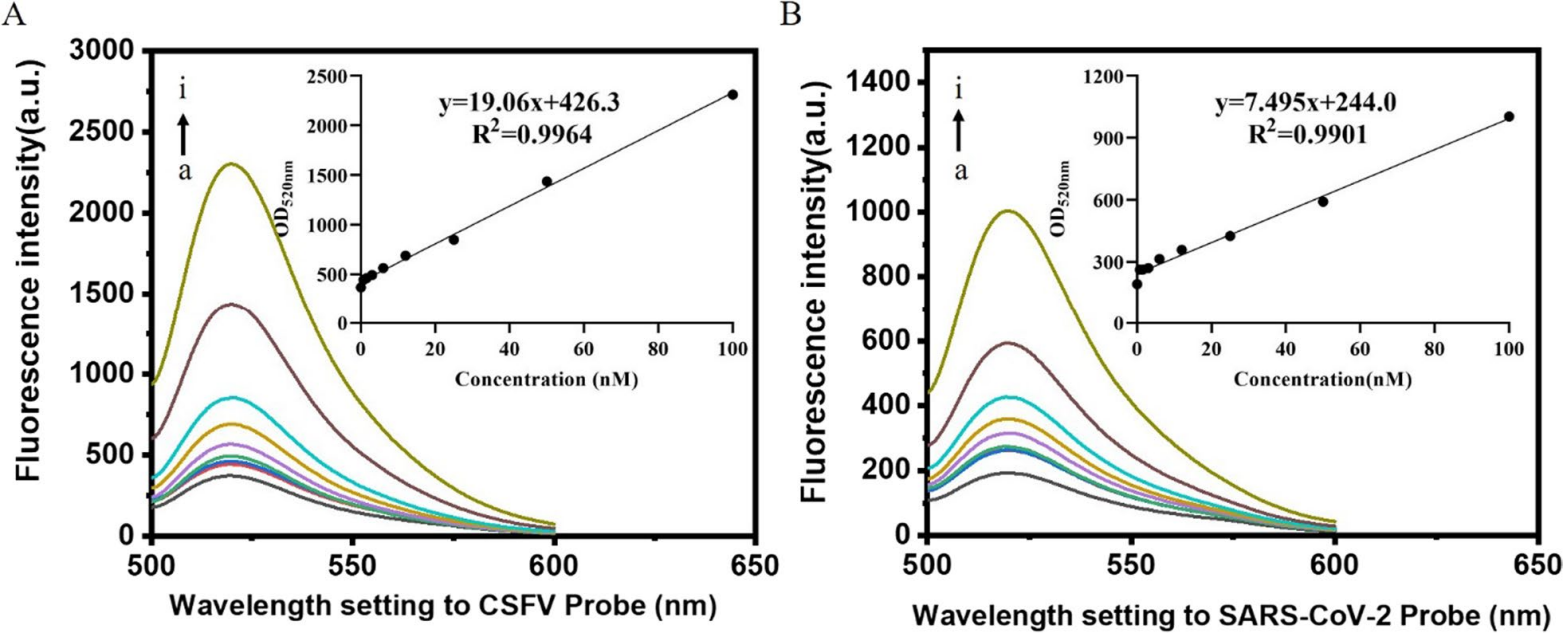
Sensitivity and specificity of CSFV and SARS-CoV-2 probes. **A** Fluorescence intensity of the strategy for the assay of CSFV target at different concentrations (a to i, 0 nM, 0.75 nM, 1.5 nM, 3 nM, 6 nM, 12 nM, 25 nM, 50 nM, and 100 nM). **B** Fluorescence intensity of the strategy for the assay of SARS-CoV-2 target at different concentrations (a to i, 0 nM, 0.75 nM, 1.5 nM, 3 nM, 6 nM, 12 nM, 25 nM, 50 nM, and 100 nM). The results are representative of three independent experiments, and data is expressed as mean ± SEM

High sensitivity is a most important feature of an excellent biosensor. To evaluate the sensitivity of CSFV and SARS-CoV-2 probes, we investigated the fluorescence intensity of two RNA virus probes with different target concentrations. As shown in Fig. 4A, when 0.75–100 nM CSFV targets were added to 100 nM CSFV RNA virus probe, the fluorescence signal intensity remarkably increased in a dose-dependent manner. Furthermore, from the results shown in Fig. 4B, the fluorescence intensity of the SARS-CoV-2 probe increased in a dose-dependent manner when the SARS-CoV-2 target concentration was varied from 0.75 to 100 nM. In this work, the detection limit was defined as LOD = 3 Sb/m, where LOD, Sb, and m are the limits of detection, standard deviation of the blank solution obtained in 11 replicate measurements, and slope of the calibration graph, respectively [13]. The corresponding LOD of CSFV and SARS-CoV-2 probes were calculated to be 0.28 nM and 0.24 nM, respectively.

In addition, to further assess the specificity of the CSFV or SARS-CoV-2 probe, probe sequences were aligned in NCBI. Blast analysis indicated that the sequence of the recognition strand was specific to CSFV or SARS-CoV-2 epidemic strains (parts of them listed in Additional file 1: Table S1 and Table S2), and no blast results were obtained on other viruses in NCBI (https://blast.ncbi.nlm.nih. gov/Blast.cgi). Meanwhile, no significant fluorescence was observed in samples containing other viruses (PCV, PRV, and PRRSV (Additional file 1: Fig. S2). Similarly, a series of targets covering different N gene sequences with high sequence similarity to SARS-CoV-2, including severe acute respiratory syndrome coronavirus (SARS-CoV), middle east respiratory syndrome coronavirus (MERS-CoV), hepatitis C virus (HCV), influenza A virus (H1N1) and zaire ebola virus (ZEBOV), were detected with SARS-CoV-2 probe, and no significant fluorescence was obtained (Additional file 1: Fig. S3, S4, Table S3). This suggests that the strategy proposed in this study is highly sensitive and specific.

Interpreting some virus strains in the Additional file 1: Table S1, the previous results showed that CSF0705 “Margarita” is a medium to highly pathogenic CSFV strain, which directly resulted in an epidemic in western Cuba in 1993 [14]. Further, a challenge strain CSFV VN91 was isolated from infected pigs in Vietnam [15], which had previously caused outbreaks of CSF in Vietnam. The field isolate ‘‘Pinar del Rio’’ (CSF1058) showed low virulence, and only caused mild symptoms and slightly elevated rectal temperatures, making it difficult to implement clinical diagnosis [16]. ‘‘Pinar del Rio’’ (CSF1058) caused two outbreaks in 1993 and 1997, as well as devastating epidemics over the past two decades in Cuba [14, 17, 18]. In Additional file 1: Table S2, ‘WH-Human 1’ coronavirus (NC_045512.2) is a new RNA virus strain from the family Coronaviridae, which was identified in China [19]. From Table S2, we further noticed several notable variants of SARS-CoV-2 that emerged in late 2020. The World Health Organization has currently declared the Alpha, Beta, Gamma and Delta SARS-CoV-2 variants as variants of concern. The transmissibility of the Alpha variant (lineage B.1.1.7) has been found to be substantially higher than that of pre-existing SARS-CoV-2 variants in the United Kingdom, Denmark, Switzerland, and the United States [20]. The Beta variant, also known as lineage B.1.351, was first detected in South Africa and reported by the country’s health department. It has been reported that, compared to other variants, the prevalence of this variant was higher among young people with no underlying health conditions, frequently resulting in serious illness in such cases [21]. The Lambda variant, also known as lineage C.37, first detected in Peru in August 2020, is a growing epidemiological threat in several South American countries, which has spread to at least 30 countries worldwide [22]. Recently, the Delta variant, also known as B.1.617.2, has become the globally dominant variant, spreading to at least 185 countries. On June 3, 2021, Public Health England reported that this strain could spread almost twice as fast as the Alpha variant [23, 24]. Very recently, the Omicron variant, also known as B.1.1.529, was firstly tested in the collection of samples in Botswana and South Africa on November 2021 [25]. Furthermore, the first confirmed case of Omicron in the United States was identified in California on December 2021, which has multiple mutations in the S protein compared with normal SARS-CoV-2 [26]. Our probe design on N protein ensures perfect specificity and coverage of detection. Thus, the blast analysis suggests that the proposed RNA virus probe could be applied for nucleic acid testing of different CSFV or SARS-CoV-2 strains derived from various locations worldwide.

### Probes in live-cells fluorescence signals

**Fig. 5:**
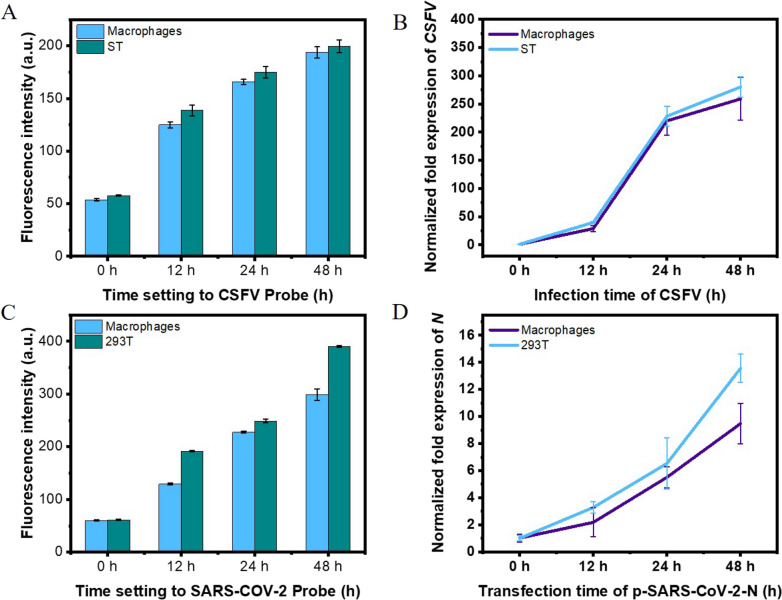
Results of fluorescence signals on RNA virus probes in live-cells were compared with qRT-PCR analysis. **A** Changes of fluorescence intensity of CSFV on cells. **B** The results of CSFV E2 gene qRT-PCR were compared with it. **C** Changes of fluorescence intensity of SARS-CoV-2-N on cells. **D** The results of SARS-CoV-2 N gene qRT-PCR were compared with it

To detect the effect of the CSFV or SARS-CoV-2 probe in in vitro biological samples, the CSFV Shimen strain at an MOI of 5 was infected into swine testicular (ST) cells and porcine alveolar macrophages 3D4/21 for 0, 12, 24, and 48 h. After the conditioned media were collected, the CSFV probe was added to the cell lysate to monitor any changes in fluorescence intensity. Figure 5A shows that the signal increased significantly in a time-dependent manner when the probe was in the cell lysate of CSFV-infected ST cells or macrophages. The fluorescence data indicated that CSFV finished their replication with the increase of time. This result is fully consistent with quantitative reverse transcription polymerase chain reaction (qRT-PCR) analysis of the CSFV Shimen strain proliferating in ST cells or macrophages (Fig. 5B). Further, we transfected 293T cells or RAW264.7 cells with pEOFP-N1-N plasmid, for 0, 12, 24, and 48 h. The results of fluorescence measurements (Fig. 5C) showed that the signal increased significantly in a time-dependent manner in both 293T cells and RAW264.7 cells. Similar results were obtained when qRT-PCR analysis was carried out with the same samples in which pEOFP-N1-N plasmids were transfected (Fig. 5D).

### Visualization of CSFV or SARS-CoV-2 in infectious cells using specific probes

**Fig. 6:**
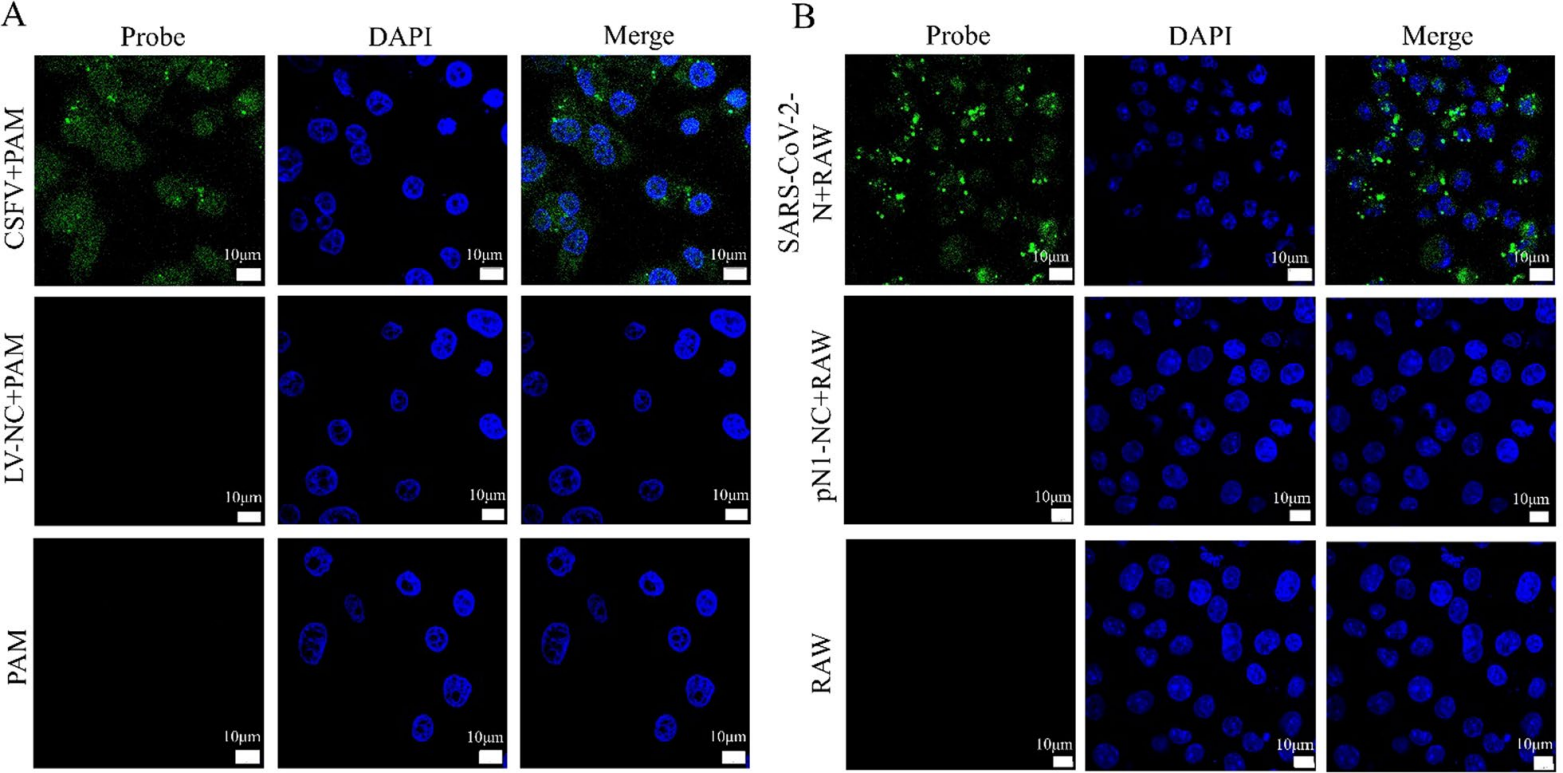
Visualization of CSFV or SARS-CoV-2 probe entering cells using live-cell fluorescence analysis. **A** Confocal microscopy analysis of CSFV probe into PAM cells infecting by CSFV. Visualization of CSFV or SARS-CoV-2 probe entering cells using live-cell fluorescence analysis. **A** Confocal microscopy analysis of CSFV probe into PAM cells infecting by CSFV. LV-NC + PAM is the mock infection group to CSFV probe into PAM cells. The PAM are the negative control experiments without CSFV infection. The probe was marked with FAM with green color and the cellular nucleus was stained blue (DAPI). The merged image clearly revealed the co-incubation of CSFV probe with PAM cells. **B** Confocal microscopy analysis of SARS-CoV-2 probe into RAW264.7 cells transfecting by plasmid including SARS-CoV-2 N gene. The pN1-NC + RAW is as mock infection to SARS-CoV-2 probe into RAW cells by pN1-NC transfection. The RAW are the negative control experiments without the pEOFP-N1-N plasmid transfection containing the SARS-CoV-2 N gene. The probe was marked with FAM with green color and the cellular nucleus was stained blue (DAPI). The merged image clearly revealed the co-incubation of SARS-CoV-2 probe with RAW264.7 cells. CSFV, classical swine fever virus; SARS-CoV-2, severe acute respiratory syndrome coronavirus 2; PAM, porcine macrophage cells; RAW, RAW264.7 cells

Confocal microscopy was used to visually assess whether CSFV or SARS-CoV-2 probes could identify the nucleotide sequences of RNA viruses in live cells. After CSFV Shimen or pEOFP-N1-N plasmids were incubated for 24 h, macrophages were treated with CSFV or SARS-CoV-2 probes, and cellular localization was tracked. As shown in Fig. 6A, confocal laser scanning microscopy revealed numerous green fluorescent signals in CSFV-transfected macrophages compared to uninfected controls. Similar results were observed in the macrophage model used to simulate SARS-CoV-2 infection (Fig. 6B). Macrophages protect the host from infection with pathogenic microorganisms and act as the frontline of immune defense [27]. However, CSFV can invade macrophages via the caveolin-1-mediated endocytic pathway, and other unknown mechanisms [28]. Recently, it was found that macrophages play an important role in COVID-19, and are located at the center of the pathological regulation of SARS-CoV-2 induced severe pneumonia cytokine storm [29]. More importantly, SARS-CoV-2 breaks the balance of the host’s immune microenvironment by infecting macrophages, resulting in damage to the host’s lungs and triggering other anti-inflammatory microenvironment disorders, eventually leading to the death of the host [30]. Our findings suggest that CSFV or SARS-CoV-2 probe act as a sensitive visual detector of the invasion of RNA virus into macrophages.

### Probes in tissue fluorescence signals

**Fig. 7:**
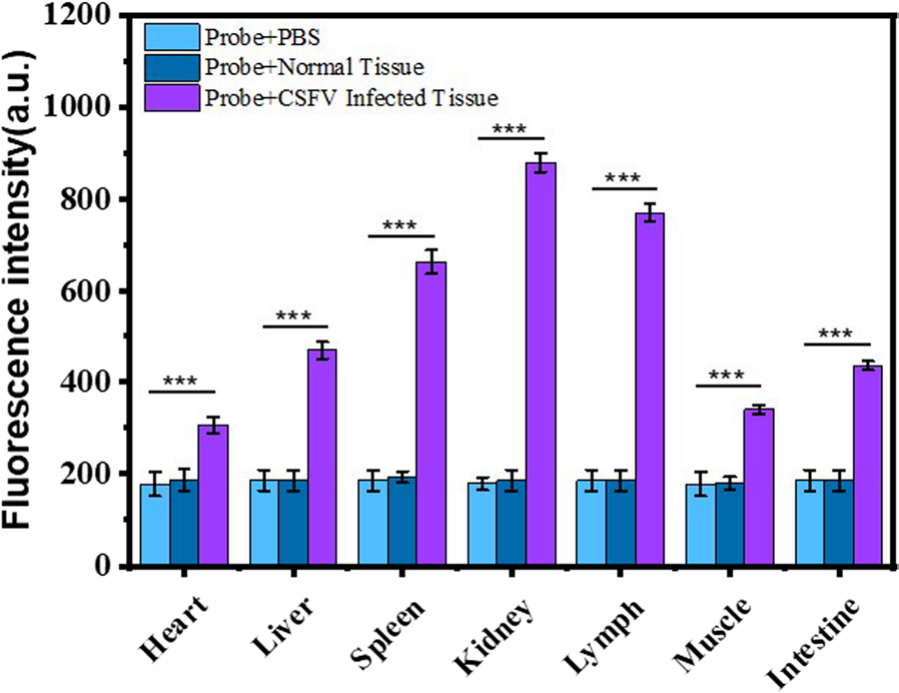
Fluorescence intensities of major organs from a normal and CSF-positive pig. The CSFV was detected at tissue level with CSFV probes. The fluorescence measurements indicated CSFV infection of CSF-positive pig and different distribution of CSFV among major organs. No significant enhanced signals were found on normal tissue

**Fig. 8:**
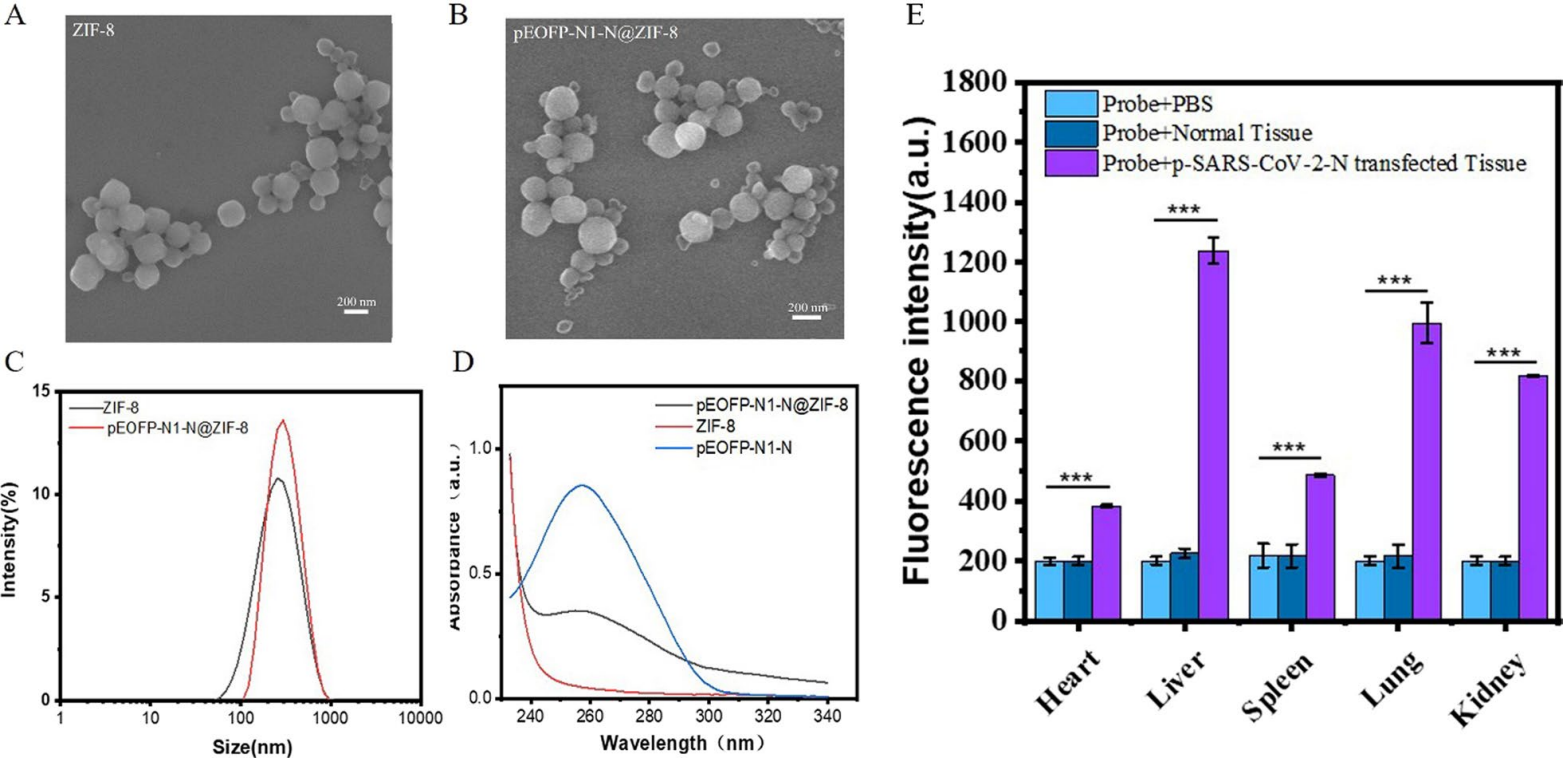
Fluorescence intensities of major organs from SARS-CoV-2-N transfected mice. A SARS-CoV-2 infected mouse model with p-SARS-CoV-2-N@ZIF-8 were established. **A** SEM characterization of ZIF-8 nanoparticles; **B** SEM characterization of pEOFP-N1-N@ZIF-8 nano-composites; **C** The size distributions of ZIF-8 and pEOFP-N1-N@ZIF-8 particles in PBS (10 mM, pH 7.4). **D** UV-vis absorption spectra of nanoparticles among ZIF-8, pEOFP-N1-N and pEOFP-N1-N@ZIF-8 particles, which support pEOFP-N1-N has been coated into ZIF-8. **E** The fluorescence measurements on SARS-CoV-2-N were detected at tissue level. The fluorescence signals indicated that different distribution of SARS-CoV-2-N gene among major organs. No significant enhanced signals were found on normal tissue

To further support the proof-of-concept for the detection technology platform, we tested the CSFV probes in seven tissue samples, namely the heart, liver, spleen, kidney, lymph, large intestine, and muscle, obtained from a CSFV-infected pig with pathologically confirmed CSF. The fluorescence measurements by CSFV probes indicated CSFV infection after standardized tissue grinding of these seven samples, while no significant difference in signal was found between the phosphate buffer solution (PBS) and control samples obtained from the CSF-negative pig (Fig. 7). Importantly, we noticed a significant difference in the fluorescence data among the seven CSFV infected tissue samples, with the highest fluorescence in the kidney sample, followed by the lymph sample. These results are consistent with those of previous reports on the distribution of CSFV [31]. The qPCR assay further supported this conclusion (Additional file 1: Fig. S5). Subsequently, a SARS-CoV-2 mouse model infected with p-SARS-CoV-2-N@ZIF-8 was established. The results from SARS-CoV-2 probe detection showed that the fluorescence signal was significantly higher in the SARS-CoV-2 infected mouse model than in the mock-infected and PBS groups. Figure 8 shows that the signals from the liver, lung, and kidney were more enhanced compared those obtained from the heart and spleen, which was in accordance with the distribution characteristics of SARS-CoV-2 nucleic acid spread by ZIF-8 [32].

Currently, RNA virus epidemics continue to wreak havoc in human society. The ongoing COVID-19 pandemic has yielded an urgent demand for rapid diagnostic testing. In addition to animal husbandry, qRT-PCR assays are recommended as the major diagnostic testing methods for RNA viruses. However, qRT-PCR assays require two steps to detect viral RNA: RNA extraction, followed by qRT-PCR amplification of the extracted RNA [33, 34], being time-consuming and requiring specific laboratory conditions [35], which produced a primary challenge to expend SARS-CoV-2 on-the-spot determination. Besides, qRT-PCR detection equipment is usually more expensive, and the operators need professional training [36]. Hence, it is essential to develop fast and economical nucleic acid detection technology to match rapid point-of-care (POC) diagnostic requirements. In our work, an extraction-free and amplification-free RNA detection method with CSFV and SARS-CoV-2 probes was successfully verified. Due to the safety limitations of SARS-CoV-2 research, the effect of the probe on SARS-CoV-2 infection in vivo could not be determined. However, different pathological tissue samples from a CSF-positive pig were verified using a linear molecular probe without the need for viral nucleic acid amplification. Although the viral probes didn’t show as much fluorescence amplification folds as those of qRT-PCR assays (Fig. 5), the obtained significant difference compared with the negative control was sufficient to support the determination of virus-positive. Considering the risk of the movement of virus-positive cases to epidemic prevention and control, it is a priority to expand point-of-care rapid virus testing when performing large-scale screening. The proposed linear molecular probe could thus be used as a universal platform for target RNA virus detection, which possesses great potential for fast, accurate, and specific measurement of different RNA viruses, facilitating the development of biomedical research and early clinical diagnosis.

## Conclusions

In summary, we developed a nucleic acid extraction-free and amplification-free RNA virus probe for the sensitive and simple detection of CSFV and SARS-CoV-2 using a double-stranded molecular beacon in combination with a quenched fluorophore. The proposed RNA virus probe comprises a complementary probe that targets a specific sequence of RNA viruses. The method displayed a wide linear range of detection (0.75–100 nM), and the CSFV and SARS-CoV-2 probes permitted detection of RNA at concentrations as low as 0.28 nM and 0.24 nM, respectively. The results were supported by conventional quantitative (qRT-PCR) and visualization (confocal microscopy) analyses. Furthermore, CSF-positive swine samples and simulated SARS-CoV-2 infected mouse samples were used to validate this detection strategy. In conclusion, the low cost and high efficacy of RNA virus probes could be expected to provide a rapid point-of-care (POC) diagnostic platform for early mass virus screening.

## Experimental section

### Probe preparation

The oligonucleotides designed in this study were synthesized by Shanghai Sangon Biotechnology Co., which were purified using HPLC and confirmed using mass spectrometry. The two oligonucleotides were hybridized at a 1:1.5 volume ratio (probe A: probe B) in PBS, which was heated to 75 °C for 30 min, and cooled slowly to 4 °C for further use.

### Detection of target DNA

All target DNA sequences and probes were diluted to a suitable concentration using PBS buffer, to produce different target DNA and probe concentrations, and the target DNA and probe were added at a 1:1.5 volume ratio into the reaction tube. The total volume of the solution in the reaction tube was 200 µL, and the target DNA and probe were mixed at 37 °C and incubated for 30 min. In this study, fluorescence signals were obtained using an F-7000 FL spectrophotometer. A fluorescence excitation light wavelength (λex) of 470 nm was used, and the sample fluorescence was scanned in the range 500–700 nm with 10 nm increments; emission peaks were recorded at 520 nm.

### Sensitivity and specify of probe assays

CSFV and SARS-CoV-2 probes were detected in the presence of different concentrations of the target DNA. PBS buffer containing 100 nM CSFV or SARS-CoV-2 virus probe were prepared, and the samples were transferred to different Eppendorf tubes and supplemented with different concentrations of targets (final concentrations of 0.75, 1.5, 3, 6, 12, 25, 50, and 100 nM). After incubation for 30 min at 37 °C, the fluorescence of the samples was measured and analyzed using the same detection protocol as described above. The specificity of CSFV and SARS-CoV-2 probes was determined using BLAST analysis with online software from NCBI (https://blast.ncbi.nlm.nih.gov/Blast.cgi).

### Cell culturing, viral gene transfection, and probe detection

Porcine testicular cells (ST), porcine macrophage cells (PAM), 293T cells, or RAW264.7, were obtained from the American Type Culture Collection (ATCC, Manassas, VA), and cultured in Dulbecco’s modified Eagle medium, high-glucose medium (HyClone, USA), or RPMI-1640 medium containing 10% fetal bovine serum in an incubator at 37 °C and 5% CO_2_, respectively. PBS (20 mM, pH 7.0) was used throughout the experiment. The CSFV Shimen strain was added to the cultures at a multiplicity of infection (MOI) of 5 when ST or PAM were 70–80% confluent. The 293T cells or RAW264.7 cells were successfully transfected with the pEOFP-N1-N plasmid containing the SARS-CoV-2 N gene. Cell samples obtained at 0, 12, 24, and 48 h after viral gene transfection were incubated for 30 min at 37 °C to allow the probe-target binding reaction, respectively.

### qRT-PCR analysis

Specific oligonucleotide primers (5′-GATCCTCATACTGCCCACTTAC-3′ and 5′-GTATACCCCTTCACCAGCTTG-3′) were used to detect CSFV [37], while the mRNA level of SARS-CoV-2-N was detected using the following specific oligonucleotide primers: 5′-GGGAACTTTGGCGATCAGGA-3′ and 5′-AGCTTAATGGCGCCTGTGTA-3′. After viral gene transfection, cellular RNA was extracted at 0, 12, 24, and 48 h. qPCR was carried out as described in our previous work, in which the qPCR thermal cycler program was 95 °C for 2 min, 40 cycles at 95 °C for 15 s, 60 °C for 31 s, and a final extension at 68 °C for 30 s [38]. An Applied Biosystems 7300 Real-Time PCR System (Applied Biosystems, USA) was used to complete the reaction.

### Confocal microscopy imaging of cells with probes

The probe transfection solution for confocal analysis was obtained using two mixture: one contained 10 µL Lipofectamine 2000 reagent and 140 µL Opti-MEM, allowed to stand for 5 min after full mixing, whereas the other was supplemented with the appropriate concentrations of detection probes to a total volume of 150 µL Opti-MEM. The probe transfection liquid solution (300 µL total volume) was finally formed when the two solutions were kept static for 15 min after full mixing. Subsequently, the cells were incubated with the prepared corresponding probe transfection solution in an atmosphere of 5% CO2 and 95% air for 1 h at 37 °C. Image analysis was conducted using the standard protocol provided by laser-scanning confocal microscopy (Olympus FV1200, Japan).

### Detection of target viral gene in tissue samples

The collected swine tissue samples were flash frozen in liquid nitrogen and subsequently subjected to tissue grinding. For the SARS-CoV-2 infection model, pEOFP-N1-N@ZIF-8 nanostructures were synthesized using the following steps. First, 300 µL DNase-free pEOFP-N1-N aqueous solution (100 µg) and a 12.5 mL 2-MIM aqueous solution (237.5 mg) were mixed and stirred at room temperature for 5 min. Then, 12.5 mL (24 mg) Zn(NO_3_)_2_·6H_2_O aqueous solution was slowly added to the mixed solution, stirred, and incubated for 15 min. The solution quickly turned opaque, and the final product was centrifuged at 8000 rpm for 10 min, washed thrice, and dispersed in water for further use [39]. Further, pEOFP-N1-N@ZIF-8 (10 mg/kg) was injected into the tail vein of the mice. A SARS-CoV-2 infected mouse model was constructed based on the in vivo distribution of the ZIF-8 nanoparticle-coated SARS-CoV-2 N gene in mice. After 6 h of circulation in vivo, the mice were dissected, and their hearts, livers, spleens, lungs, and kidneys were collected, and the isolated tissues were ground.

Each sample was measured in a separate cuvette to generate a peak fluorescence response to confirm the detection of CSFV or SARS-CoV-2 probes. Measurements were performed when 10 µL of ground tissue was added to a 90 µL probe suspension. Each peak value was recorded using the same detection protocol described above (parameter settings: EX = 470 nm, EM Strat = 500 nm, EM End = 600 nm).

### Statistical analysis

Data are presented as the mean ± standard deviation (SD) of three independent experiments. Two-way analysis of variance (ANOVA) with a post-hoc test (Bonferroni’s multiple-comparison test) was used to compare and assess statistical significance among all groups. Statistical significance was set at p < 0.05.

## Supplementary Information

**Additional file 1**: **Table S1.** The CSFV probe was specific to CSFV epidemicstrains (parts of them obtained in NCBI). **Table S2.** The SARS-CoV-2 probe wasspecific to SARS-CoV-2 epidemic strains (parts of them obtained in NCBI). **Table S3. **Sequences of used N targets oligonucleotides in this work (in 5′ to 3′ direction). **Figure S1.** Stability test of thevirus-probe. Fluorescence signal of CSFV-Probe and SARS-CoV-2-Probe was recorded under different temperature conditions. (A)The fluorescence signal wasobtained by incubating the CSFV-Probe under 25 ℃, 30 ℃, 35 ℃, 40 ℃, 45 ℃ and 50 ℃ for 1 h. (B)The fluorescence signal was obtained by incubating the SARS-CoV-2-Probe under 25 ℃, 30 ℃, 35 ℃, 40 ℃, 45 ℃ and 50 ℃ for 1 h. (C)The fluorescence signal was obtained by incubating the CSFV-Probe under 25 ℃, 30 ℃, 35 ℃, 40 ℃, 45 ℃ and 50 ℃ for 24 h. (D)The fluorescence signal was obtained by incubating the SARS-CoV-2-Probe under 25 ℃, 30 ℃, 35 ℃, 40 ℃, 45 ℃ and 50 ℃ for 24 h. **Figure S2. **Determination of the specificity of CSFV probe. The samplesincluding different Pig-susceptible viruses were detected using CSFV probe. The fluorescence measurements indicated CSFV probe was specific to CSFV sample. No significant enhanced signals were found on the samples including PCV2, PRRSV and PRV. **Figure S3.** Determination of the specificity of SARS-CoV-2 probe. The different virus N targets was detected using SARS-CoV-2 probe. The fluorescence measurements indicated SARS-CoV-2 probe was specific to N gene sequences of SARS-CoV-2. No significant enhanced signals were found on the targets of SARS-CoV, MERS-CoV, HCV, H1N1 and ZEBOV. **Figure S4.** Homologous evolutionary tree of N genes of SARS-CoV, MERS-CoV, HCV, H1N1 and ZEBOV. N gene sequences of ZEBOV (GenBank: Y09358.1), H1N1 (GenBank: AF250364.2), HCV (GenBank: KC770638.1), MERS-CoV (GenBank: MZ558081.1) and SARS-CoV (GenBank:AY541755.1) were obtained from NCBI website (https://www.ncbi.nlm.nih.gov/). **Figure S5.** The qPCR assay to CSFV positive tissues, heart, liver,spleen, kidney, lymph, muscle, and intestine. CSFV positive tissues werepreviously identified using PCR method. In order to determine the relativeexpression level of CSFV E2 gene among tissues, CSFV positive tissues, heart, liver, spleen, kidney, lymph, muscle, and intestine, were detected through qPCR method, respectively. The results showed that the relatively expression of CSFV in spleen, kidney and lymph were significantly higher than that in heart.

## Data Availability

All data generated or analyzed during this study are included in the article and additional file.
