# Supplementary material for: Development and validation of a PCR-free nucleic acid testing method for RNA viruses based on linear molecular beacon probes
Source: J Nanobiotechnology. 2022 Jun 11;20:269. doi: 10.1186/s12951-022-01470-1 (PMC9187886; doi:10.1186/s12951-022-01470-1)
Supplement: Supplementary file 1 — Additional file 1: Table S1. The CSFV probe was specific to CSFV epidemicstrains (parts of them obtained in NCBI). Table S2. The SARS-CoV-2 probe wasspecific to SARS-CoV-2 epidemic strains (parts of them obtained in NCBI). Table S3. Sequences of used N targets oligonucleotides in this work (in 5′ to 3′ direction). Figure S1. Stability test of thevirus-probe. Fluorescence signal of CSFV-Probe and SARS-CoV-2-Probe was recorded under different temperature conditions. (A)The fluorescence signal wasobtained by incubating the CSFV-Probe under 25 ℃, 30 ℃, 35 ℃, 40 ℃, 45 ℃ and 50 ℃ for 1 h. (B)The fluorescence signal was obtained by incubating the SARS-CoV-2-Probe under 25 ℃, 30 ℃, 35 ℃, 40 ℃, 45 ℃ and 50 ℃ for 1 h. (C)The fluorescence signal was obtained by incubating the CSFV-Probe under 25 ℃, 30 ℃, 35 ℃, 40 ℃, 45 ℃ and 50 ℃ for 24 h. (D)The fluorescence signal was obtained by incubating the SARS-CoV-2-Probe under 25 ℃, 30 ℃, 35 ℃, 40 ℃, 45 ℃ and 50 ℃ for 24 h. Figure S2. Determination of the specificity of CSFV probe. The samplesincluding different Pig-susceptible viruses were detected using CSFV probe. The fluorescence measurements indicated CSFV probe was specific to CSFV sample. No significant enhanced signals were found on the samples including PCV2, PRRSV and PRV. Figure S3. Determination of the specificity of SARS-CoV-2 probe. The different virus N targets was detected using SARS-CoV-2 probe. The fluorescence measurements indicated SARS-CoV-2 probe was specific to N gene sequences of SARS-CoV-2. No significant enhanced signals were found on the targets of SARS-CoV, MERS-CoV, HCV, H1N1 and ZEBOV. Figure S4. Homologous evolutionary tree of N genes of SARS-CoV, MERS-CoV, HCV, H1N1 and ZEBOV. N gene sequences of ZEBOV (GenBank: Y09358.1), H1N1 (GenBank: AF250364.2), HCV (GenBank: KC770638.1), MERS-CoV (GenBank: MZ558081.1) and SARS-CoV (GenBank:AY541755.1) were obtained from NCBI website (https://www.ncbi.nlm.nih.gov/). Figure S5. The qPCR assay to CSFV positive tissue [file 12951_2022_1470_MOESM1_ESM.docx]

**Additional file 1**

**Table S1** **the CSFV probe was specific to CSFV epidemic strains (parts of them obtained in NCBI)**

| Strain | Gene | GenBank | Identities(%) | Gaps(%) |
| --- | --- | --- | --- | --- |
| CSF0705  VN91  CSFV/1.4/dp/CSF1058/2010/Pinar  CSF1058 Pinar del Rio  CSFV-CSF1072 | E2  E2  E2  E2  E2 | JX028201.1  LC374604.1  KX576461.1  JX028204.1  MK026463.1 | 100  100  100  100  100 | 0  0  0  0  0 |
| CSF0743 | E2 | MK026456.1 | 100 | 0 |
| CSF0741 | E2 | MK026454.1 | 100 | 0 |
| CSF0740 | E2 | MK026453.1 | 100 | 0 |
| CSF0738 | E2 | MK026451.1 | 100 | 0 |
| NS9811 | E2 | MH548919.1 | 100 | 0 |
| JJ98104 | E2 | MH548918.1 | 100 | 0 |
| YI97004 | E2 | MH548916.1 | 100 | 0 |
| YI88016 | E2 | MH548915.1 | 100 | 0 |
| YI87099 | E2 | MH548914.1 | 100 | 0 |
| PdR2016 | E2 | LT985811.1 | 100 | 0 |
| IVRI/Std\|India | E2 | MK405703.1 | 100 | 0 |
| EC_PE2_JB2p | E2 | KX586773.1 | 100 | 0 |
| YI9908 | E2 | KT716271.1 | 100 | 0 |
| KPP/93 | E2 | LC016722.1 | 100 | 0 |
| JJ9811 | E2 | KF669877.1 | 100 | 0 |
| LK-VNIVViM | E2 | KM522833.1 | 100 | 0 |
| 31719#1 | E2 | MZ643988.1 | 100 | 0 |
| CSF1057 Santiago de Cuba | E2 | JX028203.1 | 100 | 0 |
| CSF1056 | E2 | JX028202.1 | 100 | 0 |
| CSF0650 | E2 | JX028200.1 | 100 | 0 |
| CSF0947 | E2 | JQ411588.1 | 100 | 0 |
| CSF0306 | E2 | JQ411570.1 | 100 | 0 |
| IVRI-CSF-BS | E2 | MT586134.1 | 100 | 0 |
| CSF-IVRI-BS | E2 | MN695315.1 | 100 | 0 |
| CSFV-JY-2010 | E2 | HQ380243.1 | 100 | 0 |
| CSFV-SS-2010 | E2 | HQ380242.1 | 100 | 0 |

**Table S2** **the SARS-CoV-2 probe was specific to SARS-CoV-2 epidemic strains (parts of them obtained in NCBI)**

| Strain | Gene | GenBank | Identities (%) | Gaps (%) |
| --- | --- | --- | --- | --- |
| SARS-CoV-2/human/AUS/A12-WW-209C/2021  SARS-CoV-2/human/IND/Wuhan/2021 | N  N | OL869974.1  OK067257.1 | 100  100 | 0  0 |
| SARS-CoV-2_B.1.1.7_VeroE6_210226_P2/2021  SARS-CoV-2_B.1.351_VeroE6_210226_P4/2021  SARS-CoV-2_B.1.351_VeroE6_210226_P4/2021  SARS-CoV-2JPN/B.1.617.2 Delta variant/2021  SARS-CoV-2/human/BRA/PR-HF116/2021  SARS-CoV-2/Felis catus/PER/UPCH_sc2_cat1/2021 | N  N  N  N  N  N | MZ314997.2  MZ314998.1  MZ314998.1  OK091006.1  MZ477758.1  MZ496614.1 | 100  100  100  100  100  100 | 0  0  0  0  0  0 |
| SARS-CoV-2_B.1.1_VeroE6_210226_P12/2020 | N | MZ314996.2 | 100 | 0 |
| hCoV-19/Japan/SZ-NIG-Y21190/2021 | N | BS001245.1 | 100 | 0 |
| SARS-CoV-2/human/DEU/FFM-UK4604/2020 | N | MW822594.1 | 100 | 0 |
| SARS-CoV-2/human/DEU/FFM-BRA1/2021 | N | MW822593.1 | 100 | 0 |
| SARS-CoV-2/human/DEU/FFM-ZAF1/2021 | N | MW822592.1 | 100 | 0 |
| SARS-CoV-2/human/PHL/COVID58620/2021 | N | MZ068161.1 | 100 | 0 |
| SARS-CoV-2/human/PHL/COVID58693/2021 | N | MZ068160.1 | 100 | 0 |
| SARS-CoV-2/human/PHL/COVID53909/2021  SARS-CoV-2/human/PHL/COVID53656/2021 | N  N | MZ068155.1  MZ068154.1 | 100  100 | 0  0 |
| SARS-CoV-2/human/USA/AZ-CDC-QDX27790466/2021 | N | OK067185.1 | 100 | 0 |
| SARS-CoV-2/human/USA/FL-CDC-QDX27830704/2021 | N | OK067184.1 | 100 | 0 |
| SARS-CoV-2/human/USA/FL-CDC-QDX27788187/2021 | N | OK067183.1 | 100 | 0 |
| SARS-CoV-2/human/USA/NY-CDC-QDX27830655/2021 | N | OK067181.1 | 100 | 0 |
| SARS-CoV-2/human/USA/PA-CDC-QDX27830609/2021 | N | OK067177.1 | 100 | 0 |
| SARS-CoV-2/human/USA/WA-CDC-QDX27830636/2021 | N | OK067174.1 | 100 | 0 |
| SARS-CoV-2/human/USA/NM-CDC-QDX27830207/2021 | N | OK067173.1 | 100 | 0 |
| SARS-CoV-2/human/USA/CO-CDC-QDX27830300/2021 | N | OK067172.1 | 100 | 0 |
| SARS-CoV-2/human/USA/FL-CDC-QDX27788225/2021 | N | OK067171.1 | 100 | 0 |
| SARS-CoV-2/human/USA/MA-CDC-QDX27788390/2021 | N | OK067170.1 | 100 | 0 |
| SARS-CoV-2/human/USA/MA-CDC-QDX27788337/2021 | N | OK067169.1 | 100 | 0 |
| SARS-CoV-2/human/USA/LA-CDC-QDX27830262/2021 | N | OK067168.1 | 100 | 0 |
| SARS-CoV-2/human/USA/FL-CDC-QDX27788204/2021 | N | OK067167.1 | 100 | 0 |
| SARS-CoV-2/human/USA/FL-CDC-QDX27788163/2021 | N | OK067165.1 | 100 | 0 |

**Table S3 Sequences of used N targets oligonucleotides in this work (in 5′ to 3′ direction)**

| Type | Sequence |
| --- | --- |
| Target-SARS-CoV N | 5′-TTGCTGCTAGACAGATTGAA-3′ |
| Target-MERS-CoV N | 5′-ATGCTGCTGCTGCTAAAAAT-3′ |
| Target-HCV N | 5′-CAACTGCTGCCCAGACCTTC-3′ |
| Target-H1N1 N | 5′-AATCTGTCTAATAGTTGGAA-3′ |
| Target-ZEBOV N | 5′-CCACAAGATCTTGACAGCAG-3′ |

**
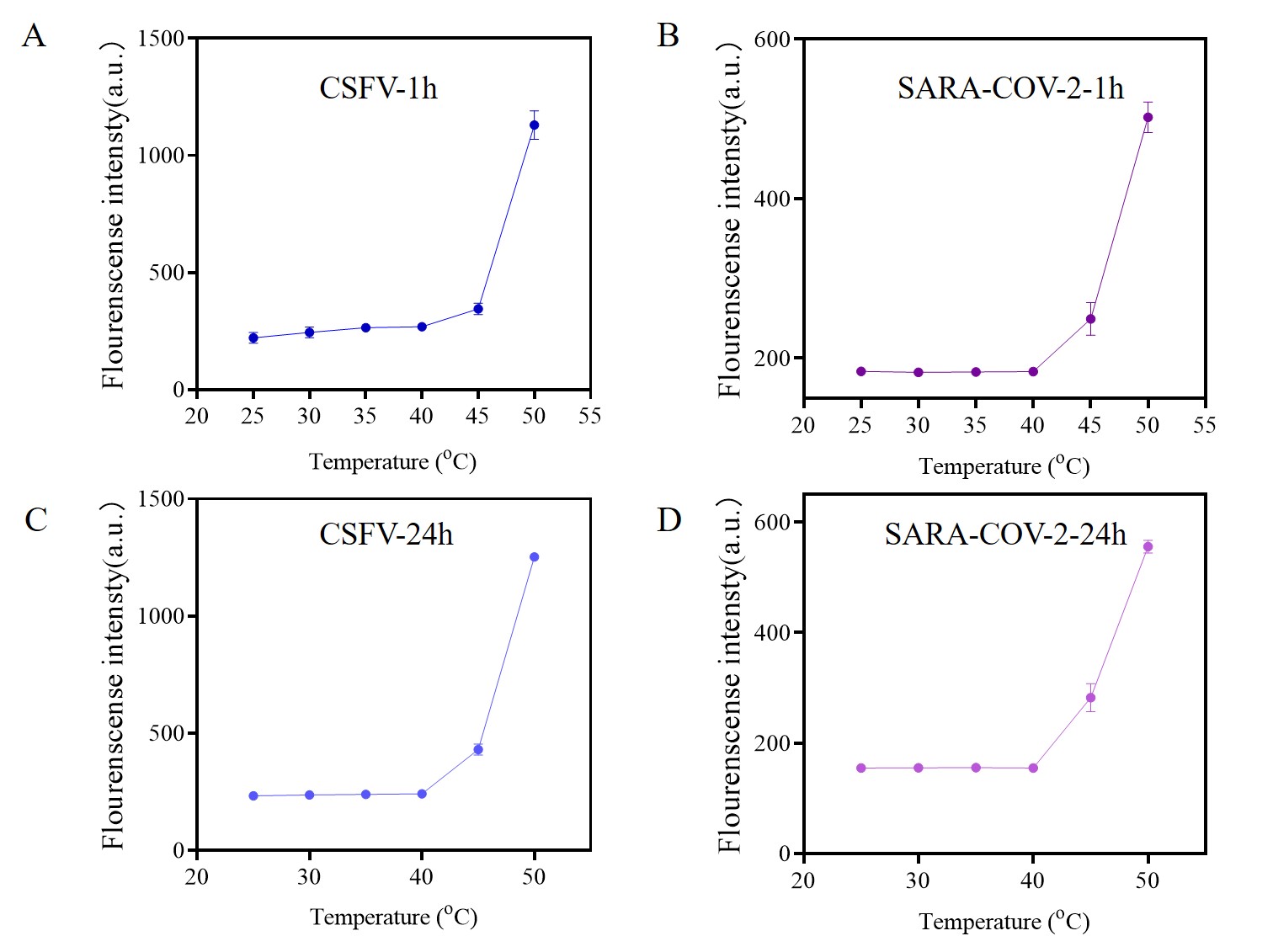
**

Figure S1. Stability test of the virus-probe. Fluorescence signal of CSFV-Probe and SARS-CoV-2-Probe was recorded under different temperature conditions. (A)The fluorescence signal was obtained by incubating the CSFV-Probe under 25℃, 30℃, 35℃, 40℃, 45℃ and 50℃ for 1 h. (B)The fluorescence signal was obtained by incubating the SARS-CoV-2-Probe under 25℃, 30℃, 35℃, 40℃, 45℃ and 50℃ for 1 h. (C)The fluorescence signal was obtained by incubating the CSFV-Probe under 25℃, 30℃, 35℃, 40℃, 45℃ and 50℃ for 24 h. (D)The fluorescence signal was obtained by incubating the SARS-CoV-2-Probe under 25℃, 30℃, 35℃, 40℃, 45℃ and 50℃ for 24 h.


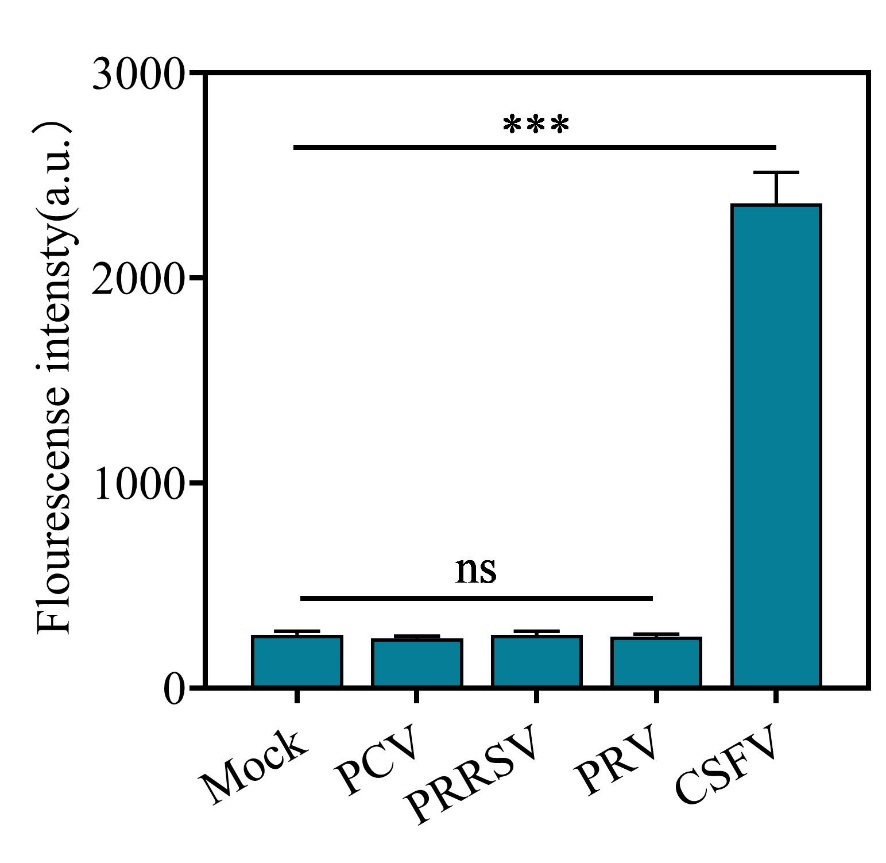


Figure S2. Determination of the specificity of CSFV probe. The samples including different Pig-susceptible viruses were detected using CSFV probe. The fluorescence measurements indicated CSFV probe was specific to CSFV sample. No significant enhanced signals were found on the samples including PCV2, PRRSV and PRV.


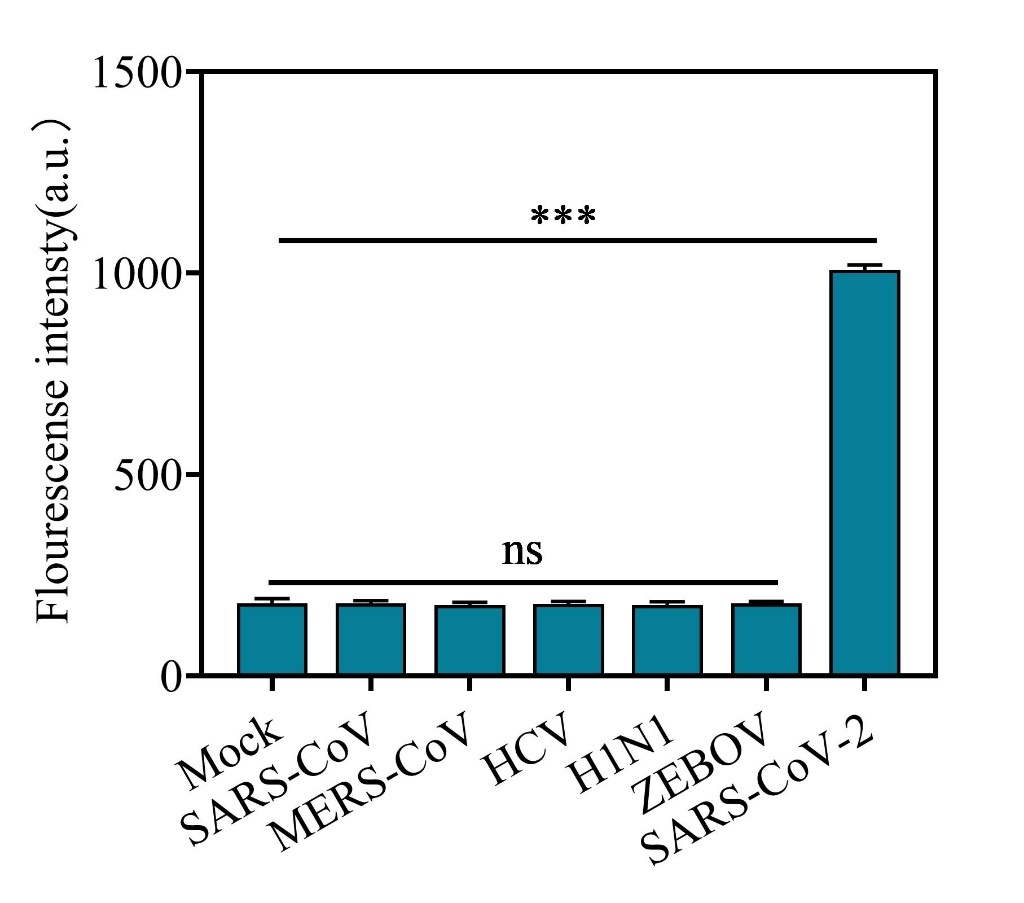


Figure S3. Determination of the specificity of SARS-CoV-2 probe. The different virus N targets was detected using SARS-CoV-2 probe. The fluorescence measurements indicated SARS-CoV-2 probe was specific to N gene sequences of SARS-CoV-2. No significant enhanced signals were found on the targets of SARS-CoV, MERS-CoV, HCV, H1N1 and ZEBOV.


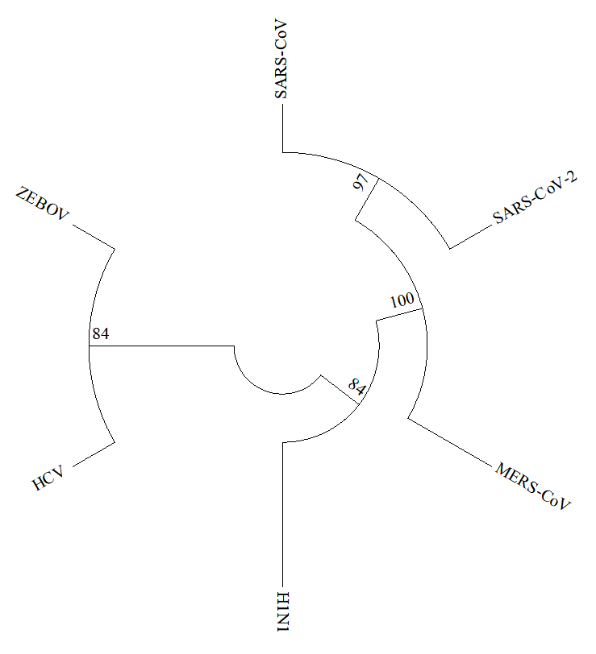


Figure S4. Homologous evolutionary tree of N genes of SARS-CoV, MERS-CoV, HCV, H1N1 and ZEBOV. N gene sequences of ZEBOV (GenBank: Y09358.1), H1N1 (GenBank: AF250364.2), HCV (GenBank: KC770638.1), MERS-CoV (GenBank: MZ558081.1) and SARS-CoV (GenBank: AY541755.1) were obtained from NCBI website (https://www.ncbi.nlm.nih.gov/).


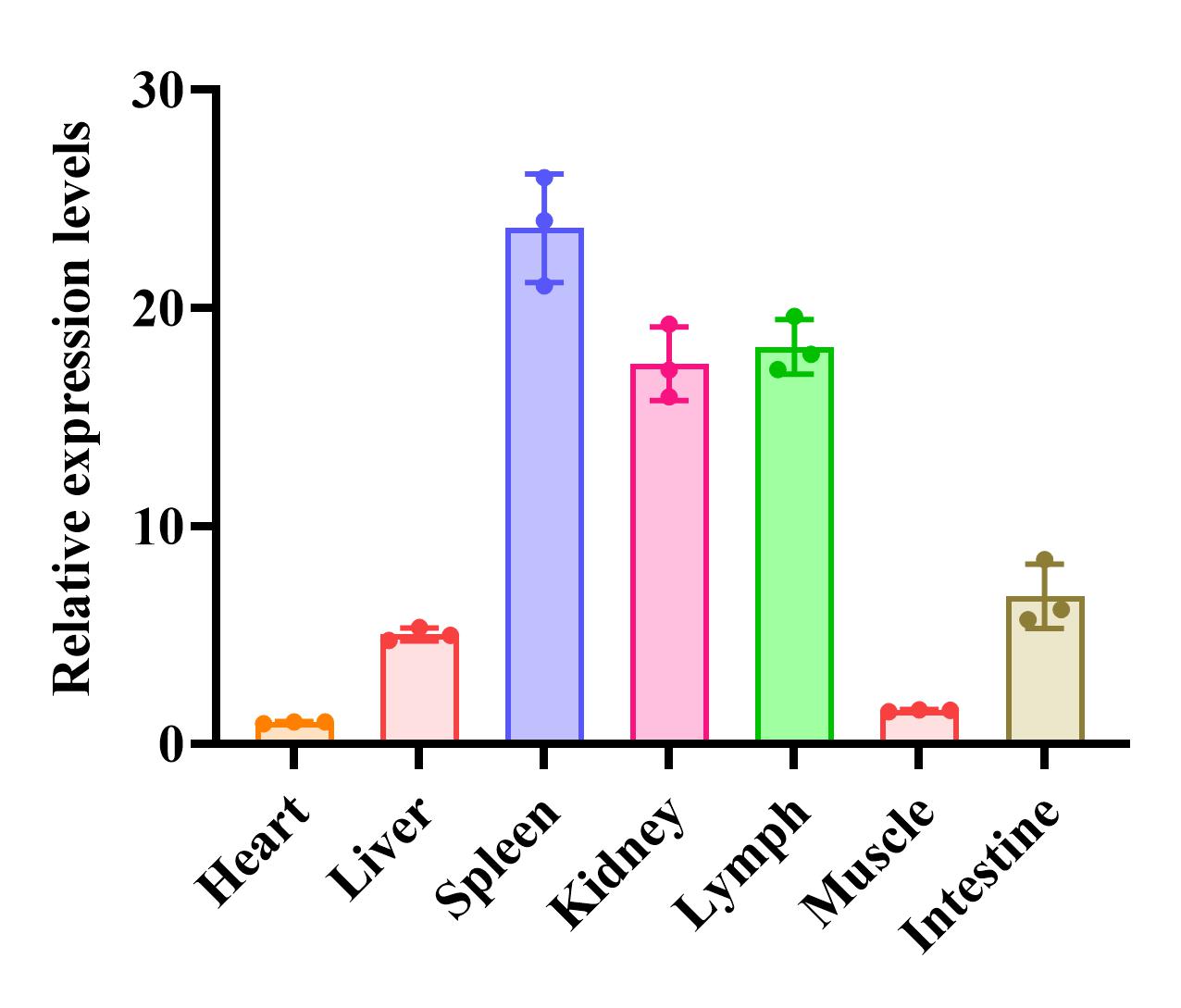


Figure S5. The qPCR assay to CSFV positive tissues, heart, liver, spleen, kidney, lymph, muscle, and intestine. CSFV positive tissues were previously identified using PCR method. In order to determine the relative expression level of CSFV E2 gene among tissues, CSFV positive tissues, heart, liver, spleen, kidney, lymph, muscle, and intestine, were detected through qPCR method, respectively. The results showed that the relatively expression of CSFV in spleen, kidney and lymph were significantly higher than that in heart.
